# A mechanistic approach to HPLC analysis, antinociceptive, anti-inflammatory and postoperative analgesic activities of panch phoron in mice

**DOI:** 10.1186/s12906-020-02891-x

**Published:** 2020-03-30

**Authors:** Zarin Tasnim Gias, Fatima Afsana, Polak Debnath, M. Shadidul Alam, Tania Naz Ena, Md Hemayet Hossain, Preeti Jain, Hasan Mahmud Reza

**Affiliations:** 1 Department of Pharmaceutical Sciences, North South University, -1229, Dhaka, Bangladesh; 2 BCSIR Laboratories, Bangladesh Council of Scientific and Industrial Research (BCSIR), -1205, Dhaka, Bangladesh

**Keywords:** Panch phoron, Antinociceptive, Anti-inflammatory, HPLC, Postoperative pain

## Abstract

**Background:**

Panch phoron is a mixture of five spices containing an equal proportion of *Foeniculum vulgare* (fennel), *Trigonella foenum-graecum Linn* (fenugreek), *Nigella sativa* (black cumin), *Cuminum cyminum* (cumin) and *Brassica nigra* (black mustard). The mixture is commonly used in Bangladeshi cuisine and possesses many pharmacological effects. In this study, we evaluated the antinociceptive and anti-inflammatory activities of aqueous panch phoron extract (PPE) in vivo, its possible mechanism of action and phytochemical analysis by High-Performance Liquid Chromatography (HPLC). We also investigated the effect of PPE on postoperative pain in mice.

**Methods:**

HPLC was carried out using LC-20A Modular HPLC system to identify the bioactive compounds present in PPE. Five groups of Swiss albino male mice (*n* = 6 per group) were orally treated with 10 ml/kg of distilled water or 10 mg/kg of sodium diclofenac or three doses of PPE (100 mg/kg, 300 mg/kg, 500 mg/kg). In vivo assessment was carried out by the writhing test, tail-flick test, formalin test, and carrageenan induced paw edema test. The opioid antagonist, naloxone was used in the acetic acid test to evaluate the involvement of opioid receptors. To assess the effect of PPE in postoperative pain, mice that underwent sciatic nerve surgery were measured for the paw withdrawal latency in a hot water bath.

**Results:**

In HPLC analysis, different types of phenolic compounds and flavonoids, including catechin hydrate, para-coumaric acid, vanillic acid, and syringic acid were detected. Treatment with PPE exhibited dose-dependent antinociceptive and anti-inflammatory activities in pain models (*p* < 0.05). Furthermore, naloxone did not reverse the effect of PPE in the writhing test. Mice that underwent sciatic nerve surgery showed that the paw withdrawal latency increased gradually over 7 days.

**Conclusions:**

Our results demonstrate that PPE has significant antinociceptive and anti-inflammatory activities and can provide significant postoperative analgesia.

## Background

Pain is an unpleasant sensory, emotional and cognitive sensation caused by various harmful stimuli which result in tissue damage and exhibit autonomic, psychological, and behavioral reactions [1]. It is a protective mechanism which causes awareness to withdraw the affected area from the tissue-damaging stimuli and to heal the site [2]. Specialized peripheral nerve fibers called nociceptors can detect thermal, mechanical or chemical stimuli and send signals to the brain and the spinal cord [1]. Conversely, postoperative pain remains a critical clinical problem resulting from various surgeries [3, 4]. Previous surveys indicate that around 74% of patients discharged from emergency wards experience moderate to severe pain, and a high percentage of cancer patients report inefficient pain management [5]. According to the World Health Organization (WHO) and the International Association for the Study of Pain (IASP), relieving from pain is undoubtedly a human right. It is necessary to manage postoperative pain to reduce complications and a long period of rehabilitation. Poorly managed acute pain may result in chronic pain, thereby decreasing the quality of life and patient satisfaction and increasing hospital stays and costs [6]. Current preventive strategies and analgesic agents such as nonsteroidal anti-inflammatory drugs (NSAIDs) and opioids have limited effectiveness and safety, and are associated with nausea, vomiting, sedation, respiratory depression, gastrointestinal lesions, renal failure, liver failure, and tolerance and addiction [3, 4, 7]. Further studies are essential to search for new and effective alternative medicines for preventative strategies and pain management. Considering the major analgesic prototypes of pain are morphine and salicylic acid, both obtained from plant sources, panch phoron has been studied here for antinociceptive, anti-inflammatory and postoperative analgesia in mice [7].

Panch phoron is a blend of five (panch) spices of equal proportion consisting of *Foeniculum vulgare* (fennel), *Trigonella foenum-graecum Linn* (fenugreek), *Nigella sativa* (black cumin), *Cuminum cyminum* (cumin) and *Brassica nigra* (black mustard). This mixture is also known as Paanch Phorana in Maithili, Pas Phorôn in Assamese and Panchu Phutana in Oriya and commonly used in cousins of Eastern India [8]. Accumulating evidence has demonstrated that each of these seeds have numerous pharmacological effects including analgesia [9, 10], anti-inflammatory [9–12], antioxidant [9, 12], cardioprotective [9], neuroprotective [9, 12], anti-diabetic [11], chemoprotective [9, 12], gastroprotective [9], and nephroprotective activities [9].

Studies showed that the presence of alpha-pinene and fenchone in *Foeniculum vulgare* exhibited analgesic activities against tail-flick test [13] whereas estragole, gallic acid and L-limestone in these seeds were responsible for antioxidant activities in mice [14]. Previously, anti-inflammatory activities of *Foeniculum vulgare* was also determined using the formalin-induced nociception test, which showed prominent analgesic activity in mice model [15]. Glycoside and steroidal components present in *Trigonella foenum-graecum Linn* exhibited analgesic and anti-inflammatory effects against acetic acid-induced writhing test in mice [16] and carrageenan-induced paw edema test in rats, respectively [12]. In a study, it was discussed that *Nigella sativa significantly reduced the number of writhing against acetic acid-induced writhing test in mice and it was also mentioned that the bioactive components, t*hymoquinone and para-benzoquinones *present in these seeds could cause antinociceptive effects by hampering the prostaglandin synthesis* [17]*.* Another study proved that *Cuminum cyminum* plant contains monoterpene compounds, such as γ-terpinene α-pinene, linalool and β-pinene, which could exert an anti-inflammatory effect in a rat model. This study also described that the inhibition of COX enzyme by *Cuminum cyminum* could be the reason for its analgesic activities against acetic acid-induced writhing test in mice [18]. A few evidence showed that *Brassica nigra*, one of the five seeds present in panch phoron could significantly reduce inflammation in arthritic rats [19].

Although individual seed exerts analgesic and anti-inflammatory activities, there is no evidence which shows a combination of these five spices can result in reducing pain, inflammation and decrease postoperative pain. Therefore, this study investigates the antinociceptive, anti-inflammatory and postoperative analgesic activities of panch phoron in mice and its underlying pharmacological mechanism.

## Materials and methods

### Chemicals

All the chemicals that were used in the study of PPE were of analytical or HPLC grade. These were either purchased from Shimadzu (Tokyo, Japan), Sigma–Aldrich (St. Louis, MO, USA), Hwashin (Korea), Sartorius (Germany) or Merck (Darmstadt, Germany).

### Seed material

The seeds of panch phoron were obtained from the local market of Dhaka, Bangladesh and authenticated by the Bangladesh National Herbarium, Zoo Road, Dhaka 1216. A voucher specimen is retained in the Herbarium as well as in the Department of Pharmaceutical Sciences, North-south University, under the specimen number of DACB 46563.

### Preparation of the extract

The seeds were dried for 2 days at room temperature and crushed into a powder which was then soaked in distilled water and kept on an extraction shaker for several days. The solution was extracted by maceration and concentrated into a mass by using a rotary evaporator under reduced pressure at 60^O^. This panch phoron crude extract was then stored in the refrigerator and subsequently refereed as PPE (panch phoron extract) in this study.

### Animals

Swiss albino male mice (25-30 g) used for experiments were obtained from the central animal house of North South University. The animals were accommodated in cages of polypropylene and maintained at constant room temperature (25+/− 2 °C), relative humidity 61–65% in a light-dark cycle of 12 h. The animals were provided with standard laboratory diet and water and they were checked every day for any health issues. This project was carried out following strict rules and regulations for the Care and Use of Laboratory Animals of the National Institutes of Health and under the ARRIVE (Animal Research Reporting In Vivo Experiments) guidelines. Animal Care and Use Committee (IACUC) of North South University (2019/OR-NSU/IACUC-No.0405) approved the experimental protocol used on laboratory animals. After the experiments, all mice were euthanized under standard protocol using pentobarbital 150 mg/kg, IP and efforts were made to minimize any suffering. The mice (*n* = 6 per group) were used for antinociceptive, anti-inflammatory activities and postoperative analgesia and divided into five different groups. Group 1, the negative control group was treated with 10 ml/kg distilled water. Group 2 served as the positive control where the mice were treated with sodium diclofenac 10 mg/kg, as the standard drug, and the next three groups of animals were given three different doses (100 mg/kg, 250 mg/kg and 500 mg/kg) of PPE. Tests were performed on separate days.

### Qualitative analysis of phytochemicals

The PPE was tested for the presence of bioactive compounds using standard qualitative methods [20]. Alkaloids in the extract were determined by Wagner’s method, whereas flavonoids were tested using magnesium chloride (MgCl_2_) and hydrochloric acid (HCl). To detect the presence of reducing sugar, Fehling’s reagent A, Fehling’s reagent B, and Benedict’s reagent were used. Phenolic compounds were tested using lead acetate test; phytosterols were studied using Liebermann Burchard test and saponins were determined by foam test. The presence of gums and mucilage were tested by the ability to form a white and cloudy precipitate with absolute alcohol and fixed oils by saponification test.

### Preparation of standard solutions for HPLC analysis

Sixteen phenolic compounds were dissolved in methanol to prepare stock standard solutions with concentrations ranged from 4.0 to 50 μg/ml. A suitable volume of each stock solution was mixed and serially diluted to produce the working standard solutions and kept in the refrigerator.

### HPLC analysis

HPLC analysis was carried out on a LC-20A with a binary solvent delivery pump (SIL-20A HT), an autosampler (SIL-20A HT), column oven (CTO-20A) and a photodiode array detector (SPD-M20A) and controlled by the LC solution software (Luna C18 (5 μm) Phenomenex column (4.6 × 250 mm) at 33 °C. The mobile phase was composed of A (1% acetic acid in acetonitrile) and B (1% acetic acid in water) with gradient elution: 0.01–20 min (5–25% A), 21–30 min (25–40% A), 31–35 min (40–60% A), 36–40 min (60–30% A), 41–45 min (30–5% A), and 46–50 min (5% A). In this analysis, the sample injection volume was 20 μL, and the flow-rate was set at 0.5 mL/min. The wavelength of the UV detector was set at 270 nm and used for validation of method and analysis. 0.45 μm nylon membrane filter was used to filter the mobile phase and then degassed under vacuum.

Calibration curve was detected by using a standard stock solution prepared in methanol containing Gallic acid (20 μg/ml); 3,4-Dihydroxy benzoic acid (15 μg/ml); Catechin hydrate (50 μg/ml); Catechol, (−) Epicatechin, Rosmarinic acid (30 μg/ml each); Caffeic acid, Vanillic acid, Syringic acid, Rutin hydrate, p-Coumaric acid, trans-Ferulic acid, Quercetin (10 μg/ml each); Myricetin, Kaempferol (8 μg/ml each); trans-Cinnamic acid (4 μg/ml). The concentration of the extract was prepared as 10 mg/ml. All the solutions were filtered properly through 0.20 μm syringe filter and degassed for 15 min in an ultrasonic bath before the analysis. Data acquisition, peak integration, and calibrations were calculated with Lab Solution software.

### Antinociceptive activity

#### Acetic acid-induced writhing test

The analgesic effect of PPE was tested by the writhing test where five different groups of mice were orally treated with distilled water (10 ml/kg) or sodium diclofenac (10 mg/kg) or three different doses of PPE (100 mg/kg, 250 mg/kg, 500 mg/kg). After 30 min, 0.2 mL of 3% acetic acid solution was injected intra-peritoneally to induce pain to all the mice of different groups. The number of writhes (abdominal constrictions) was counted and recorded that occurred between 5 to 20 min after acetic acid injection [21]. The percentage inhibition of writhing by PPE was calculated by using the following formula:

$$ \%\mathrm{analgesic}\ \mathrm{activity}=\frac{\mathrm{Mean}\ \mathrm{writhing}\ \mathrm{count}\ \left(\mathrm{control}\ \mathrm{group}-\mathrm{treated}\ \mathrm{group}\right)}{\mathrm{Mean}\ \mathrm{writhing}\ \mathrm{count}\ \mathrm{of}\ \mathrm{control}\ \mathrm{data}}\ast 100 $$

#### Tail-flick method

Five groups of mice were treated orally with distilled water (10 ml/kg) or sodium diclofenac (10 mg/kg) or three doses of PPE (100 mg/kg, 250 mg/kg, 300 mg/kg). The lower 2 cm section of the tail was dipped in a beaker of hot water in which a temperature of 50 ± 1 °C was maintained. The time interval of the tail-flick method was taken as the indication of antinociception and was determined at 0, 30, and 60 min after the administration of the distilled water, drug and extracts. The maximum reaction time was kept fixed at 10 s [22].

#### Formalin test

The formalin-induced nociception was performed in five groups of mice which were treated with distilled water or sodium diclofenac or three doses of PPE (100 mg/kg, 250 mg/kg, 300 mg/kg). After 60 min of oral treatment, mice were injected subcutaneously with 20 μL of 3% formalin into the plantar surface of the right hind paw. The mice were placed in glass beakers and observed for 30 min. The duration of licking and biting of the paw was observed for two phases- early phase (0-5 min) and late phase (15-30 min) [23]. The percentage of licking and biting inhibition was calculated by the following formula:

$$ \%\mathrm{inhibition}\ \mathrm{of}\ \mathrm{licking}=\frac{\mathrm{Mean}\ \mathrm{of}\ \mathrm{the}\ \mathrm{control}\ \mathrm{group}-\mathrm{mean}\ \mathrm{of}\ \mathrm{the}\ \mathrm{test}\ \mathrm{group}}{\mathrm{mean}\ \mathrm{of}\ \mathrm{the}\ \mathrm{control}\ \mathrm{group}}\ast 100 $$

### Anti-inflammatory activity

#### Carrageenan-induced paw edema

Anti-inflammatory effect of PPE was tested using the carrageenan-induced paw edema test where 0.1 ml of carrageenan suspension (1%) was prepared in normal saline. Following 30 min of oral administration of distilled water or sodium diclofenac or three different doses of PPE to five different groups of mice, carrageenan was injected into the sub-plantar tissue of right hind paw to induce paw edema. Acute inflammation of the paw was then measured using a plethysmometer at hour 0 (just before administering carrageenan), 1, 2, 3, 4 and 5 h [24].

### Study for the mechanism of action of PPE

#### Involvement of the opioid system

The involvement of the opioid receptors was determined by the writhing test where 2 mg/kg of naloxone was injected subcutaneously, followed by oral administration of 500 mg/kg of PPE. The result was then compared with the negative control that received 10 ml/kg of distilled water, positive control which received sodium diclofenac of 10 mg/kg and another group that had received only PPE-500 mg/kg [25, 26].

### Sciatic nerve surgery to determine the postoperative effect of PPE

#### Sciatic nerve surgery

Mice weighing between 25 and 30 g were taken for sciatic nerve surgery as described previously, [27] with slight modifications. The mice were anaesthetized using ketamine, and the sciatic nerve of the right leg was exposed at the thigh level to undergo surgery. The sciatic nerve was crushed using a pair of # 5 forceps for 10 s, followed by the closing of the incision. All mice were separately kept in polypropylene cages and maintained a standard condition with high supervision.

#### Analgesic treatments

After the surgery, the negative control group was given no postoperative analgesics, where positive control group was treated with 10 mg/kg of sodium diclofenac per day for 7 days. For the assessment of postoperative analgesia, every day, mice were treated with PPE of 500 mg/kg orally for a week. We used only PPE of 500 mg/kg dose as this showed the most prominent antinociceptive activity in the other pain models. The drug and the extract were given at noon, every day to maintain a 24-h dosage regimen. A water bath above thermoneutral temperature [28] of mice (35 °C) was used as a thermal stimulus, and the time taken to withdraw the paw of the affected leg was recorded as the paw withdrawal latency. With an interval of 2 min, each hind paw was examined three times and averaged for each animal.

### Statistical analysis

All the results in this study were expressed as means ± S.E.M. The data was analyzed using Statistical Package for the Social Sciences (SPSS) to test statistical significance by One-Way ANOVA or Repeated Measures of ANOVA, followed by Dunnett’s test as post hoc. A value of *p* < 0.05 was considered statistically significant.

## Results

### Phytochemical screening

Phytochemical screening showed that alkaloids, gums, mucilage, saponins, reducing sugar, phytosterols, phenolic compounds, and flavonoids were present in PPE.

### HPLC-DAD analysis of phenolic contents in PPE

**Fig. 1:**
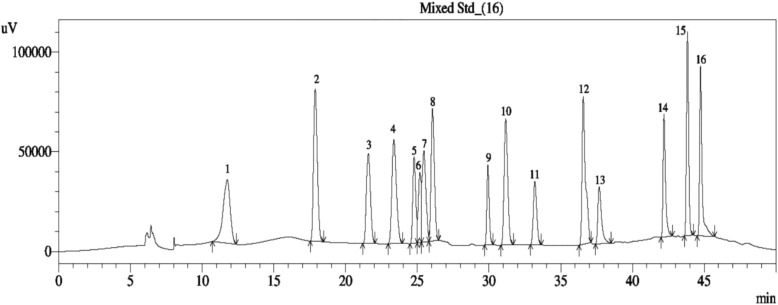
High-Performance Liquid Chromatogram (HPLC) of PPE

HPLC-DAD system was used for identification and quantification of individual phenolic compounds of PPE. Figure 1 shows polyphenolic standards containing sharp, symmetrical and well-resolved peaks that were observed in sixteen compounds. The corresponding result is given in Table 1 which indicates that PPE contains a high amount of catechin hydrate, para-coumaric acid, vanillic acid, and syringic acid. (−) Epicatechin and quercetin are also moderately present in PPE.

**Table 1:** Identification and quantification of polyphenolic compounds in PPE by HPLC analysis

Name of the Phenolic Standard	Result (mg/100 g dry extract)
1. Gallic acid	Not detected
2. 3,4 dihydroxy benzoic acid	Not detected
3. Catechin hydrate	157.783
4. Catechol	19.817
5. (−) Epicatechin	66.920
6. Caffeic acid	9.998
7. Vanillic acid	93.534
8. Syringic acid	88.742
9. Rutin hydrate	11.572
10. p-coumaric acid	94.678
11. Trans-Ferulic acid	13.098
12. Rosmarinic acid	Not detected
13. Myricetin	Not detected
14. Quercetin	43.852
15. Trans-Cinnamic acid	Not detected
16. Kaempferol	Not detected

### Acetic acid-induced writhing test

Table 2 shows the antinociceptive effect of PPE by using the writhing test in mice. The percentage of antinociception for three different doses of PPE ranges from 8.7–53.6%. PPE exhibited antinociceptive activity in a dose-dependent manner (*p* < 0.05) where the highest activity was shown by 500 mg/kg of PPE close to the percentage inhibition of sodium diclofenac (65.8%).

**Table 2:** Effect of PPE on acetic acid-induced writhing test in mice

Treatment	Dose	No of writhing	% of writhing	% of writhing inhibition
Control (distilled water)	10 ml/kg	39.2 ± 1.3	100.0	0.0
Na diclofenac	10 mg/kg	13.4 ± 1.3*	34.2	65.8
PPE	100 mg/kg	35.8 ± 4.2*	91.3	8.7
PPE	250 mg/kg	24.0 ± 0.7*	61.2	38.8
PPE	500 mg/kg	18.2 ± 1.7*	46.4	53.6
PPE (500 mg/kg) + Naloxone (2 mg/kg)		13.5 ± 0.4*	34.4	65.6

*N* = 6. Values are mean ± SEM. *p < 0.05 compared to control (One-Way ANOVA followed by Dunnett’s post hoc test)

### Tail-flick test

The antinociceptive activity of PPE was also assessed by the tail-flick test, as shown in Table 3. We observed that PPE increased the latency period in a dose-dependent manner (*p* < 0.05) indicating antinociceptive activity of the extract. The standard drug, sodium diclofenac also showed similar effects compared to the control group.

**Table 3:** Effect of PPE on tail-flick test in mice

Treatment	Dose	0 s	30 min	60 min
Control (distilled water)	10 ml/kg	3.2 ± 0.1	2.7 ± 0.6	2.4 ± 0.4
Na diclofenac	10 mg/kg	3.1 ± 0.1*	4.0 ± 0.1*	4.2 ± 0.1*
PPE	100 mg/kg	2.5 ± 0.3*	4.1 ± 0.7*	4.4 ± 0.7*
PPE	250 mg/kg	3.2 ± 0.4*	4.8 ± 0.2*	6.2 ± 0.6*
PPE	500 mg/kg	3.6 ± 0.2*	5.6 ± 0.2*	7.8 ± 0.8*

*N* = 6. Values are mean ± SEM. *p < 0.05 compared to control (Repeated measures ANOVA followed by Dunnett’s post hoc test)

### Formalin test

The anti-inflammatory activity of PPE was examined by the formalin test as shown in Table 4. The three different doses of PPE showed significant antinociceptive activity (*p* < 0.05) in both phases, where the percentage of inhibition in the early phase and late phase ranged from 40 to 65% and 73.4–80%, respectively. However, the late phase showed more prominent activity compared to the early phase. The data also showed the effect of PPE was comparable to sodium diclofenac.

**Table 4:** Effect of PPE in the early (0-5 min) and late phases (15-30 min) of the formalin test in mice

Treatment	Dose	Licking Time (sec)		% of inhibition	
		0–5 min	15–30 min	0–5 min	15–30 min
Control (distilled water)	10 ml/kg	18 ± 1.61	6.4 ± 1.03		
Na diclofenac	10 mg/kg	8.4 ± 0.51*	1.8 ± 0.37*	52.8	72.3
PPE	100 mg/kg	10.8 ± 0.86*	1.6 ± 0.24*	40.0	73.8
PPE	250 mg/kg	8.0 ± 0.32*	1.6 ± 0.24*	54.4	75.4
PPE	500 mg/kg	6.2 ± 0.37*	1.2 ± 0.20*	65.0	80.0

*N* = 6. Values are mean ± SEM. *p < 0.05 compared to control (Repeated measures ANOVA followed by Dunnett’s post hoc test)

### Carrageenan-induced paw edema

**Fig. 2:**
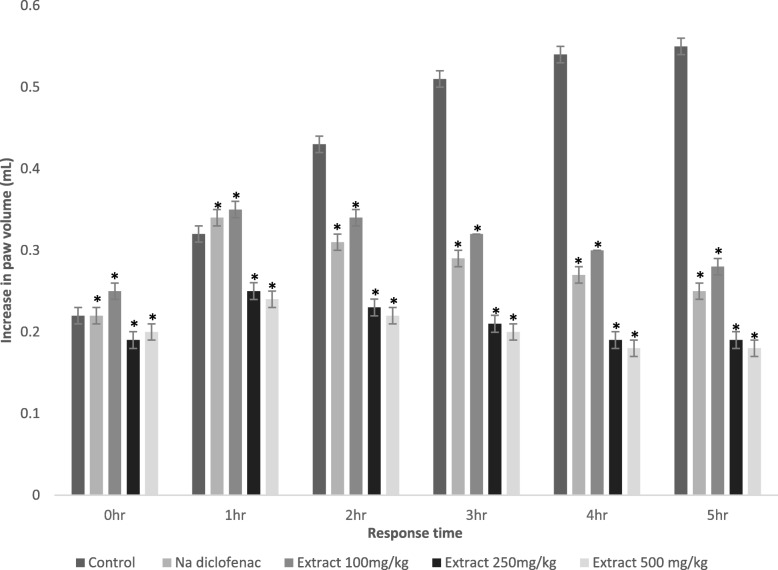
Effect of negative control (distilled water; 10 ml/kg), positive control (sodium diclofenac; 10 mg/kg) and PPE (100, 250 and 500 mg/kg) against carrageenan-induced paw edema in mice. Each column represents data in mean ± SEM of 6 mice. *p < 0.05 compared to control (One-Way ANOVA followed by Dunnett’s post hoc test)

PPE was further tested for the anti-inflammatory effect using carrageenan-induced paw edema experiment. Results are presented in Fig. 2. It was observed that three doses of PPE showed significant and dose-dependent antinociception (*p* < 0.05) for 5 h. Interestingly, we found that both 250 mg/kg and 500 mg/kg of PPE produced higher antinociceptive activity than sodium diclofenac over the period.

### Involvement of the opioid system

The results for the involvement of opioid receptors are summarized in Table 2. Pre-treatment with naloxone followed by oral administration of 500 mg/kg PPE (as this dose showed the highest percentage of writhing inhibition) inhibited 66.5% of writhing similar to sodium diclofenac (65.99%) and 500 mg/kg of PPE in the absence of naloxone (53.6%). This confirms that opioid receptors were not involved in the antinociceptive effect of PPE.

### Sciatic nerve surgery to determine the postoperative effect of PPE

**Fig. 3:**
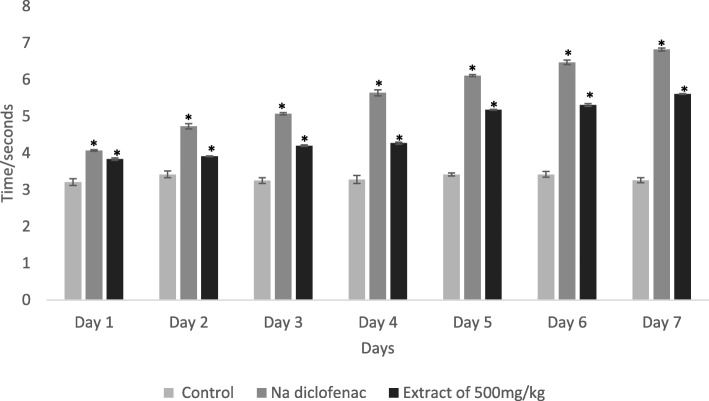
Effect of negative control (distilled water; 10 ml/kg), positive control (sodium diclofenac; 10 mg/kg) and PPE (500 mg/kg) after sciatic nerve surgery for 7 days. Each column represents data in mean ± SEM of 6 mice. *p < 0.05 compared to control (One-Way ANOVA followed by Dunnett’s post hoc test)

Figure 3 shows the data of paw withdrawal latency of the affected leg in a hot water bath after sciatic nerve surgery for a week. These data suggest that PPE increases the paw withdrawal latency gradually over 7 days which was similar to the effect of sodium diclofenac compare to the control group. Notably, a prominent effect was found in the last 3 days of the experiment for both the standard drug and PPE.

## Discussion

The study shows that oral administration of PPE exerts potent and dose-dependent antinociceptive and anti-inflammatory effects induced by chemical and thermal stimuli. It has also revealed that PPE increases paw withdrawal latency in a hot water bath after sciatic nerve surgery.

The phytochemical analysis of PPE detected the presence of alkaloids, gums, mucilage, saponins, reducing sugar, phytosterols, phenolic compounds, and flavonoids. Previous studies proved that the phenolic compounds and flavonoids present in medicinal plants could be responsible for their anti-inflammatory activity [29]. In this study, HPLC of PPE showed a significant amount of various phenolic compounds and flavonoids, thereby indicating that the extract could be useful in reducing inflammation. Catechin hydrate [30], (−) epicatechin [31] and p-coumaric acid [32] are natural phenolic compounds and flavonoids which act as potent antioxidants to scavenge free radicals in the body. Quercetin is also categorized as a flavonoid which exerts antioxidant activity, thereby useful in various anti-inflammatory diseases [33]. The antioxidant, vanillic acid is also used to alleviate inflammatory pain by hindering oxidative stress, production of cytokines and activation of NFKB in mice [34]. Syringic acid, a known antioxidant, has demonstrated to reduce pain in acute pancreatitis [35]. According to scientific data, inflammation may occur as a result of an imbalance in natural antioxidants, which lead to different inflammatory diseases [33]. Thus, PPE can be used to reduce inflammation by scavenging harming free radicals in the body.

The acetic acid-induced writhing test is a well-established experiment to investigate the peripheral analgesic activity of drugs. Evidence suggests that the pain caused by acetic acid is due to the secretion of endogenous substances and pain mediators, such as bradykinin, serotonin, substance P, histamine, prostaglandins (PGE2 and PGF2α), and proinflammatory cytokines including tumour necrosis factor-α (TNF-α), interleukin-1β (IL-1β), and IL-8 which stimulate peripheral nociceptive neurons [7, 36, 37]. Our study shows that PPE reduces the abdominal contractions of the mice in a dose-dependent manner, thereby indicating the inhibition of nociceptors activation by one of such endogenous mediators.

The tail-flick test investigates a spinal reaction which measures nociceptive response latencies to thermal stimuli and determines the central analgesic effects of drugs [38, 39]. The present study has demonstrated that sodium diclofenac and three doses of PPE alleviated the pain over 60 min to thermal stimuli. This finding suggests that PPE can inhibit central analgesic effects in the body.

Formalin test is a validated test for nociception, which gives a distinct biphasic nociceptive response. Formalin injection at the paw produces an inflammatory response resulting in swelling and licking of the paw [21, 23]. The pain caused during the early phase of the formalin test is a direct effect on nociceptors (non-inflammatory pain) whereas; the late phase is related to pain from inflammation [40]. Studies show that centrally acting drugs, such as opioids inhibit both phases equally, although the first phase is more sensitive to such substances. However, NSAIDs and corticosteroids act only in the late phase [7, 37] but, acetylsalicylic acid and paracetamol are antinociceptive in both phases [41]. Our study showed that the PPE response similarly as sodium diclofenac in the first and second phases of the formalin test, although the response is more prominent in the second phase indicating pain from inflammation.

Carrageenan is a sulphated polysaccharide acquired from seaweed which is used to determine anti-inflammatory drugs. This potent chemical induces acute inflammation and is thought to be bi-phasic. The early phase releases histamine, serotonin, and kinins in the first few hours, whereas the later phase causes inflammation by producing bradykinin, prostaglandin, and lysosome within the next 2–3 h. Evidence suggests that the second phase is sensitive to both steroidal and NSAIDs [42]. In this study, we have observed that PPE reduces paw edema over 5 h which may be due to the inhibition of cyclooxygenase-2 responsible for the downregulation of prostaglandins.

The involvement of opioid receptors by PPE was determined by using the acetic acid writhing test in the presence of naloxone. Generally, opioid receptors are responsible for centrally acting analgesics. The opioid antagonist, naloxone did not reverse the effect of PPE in mice completely which suggest that the antinociceptive effect was probably not conducted through these receptors [36] and may exert their effects by peripheral mechanism [25]. A limitation of our current study is that we could not perform the experiment for other biochemical markers such as COX-2, TNF-α, interleukins etc. which could explain the mechanistic pathway more clearly.

Studies show that sciatic nerve surgery can induce thermal hyperalgesia and mechanical allodynia, causing neuropathic pain in mice [43]. In this study, we investigated the thermal sensitivity and painful sensation of the mice that underwent sciatic nerve surgery by measuring the paw withdrawal latency of the affected leg in a hot water bath. The result showed that the latency period gradually increased within 7 days in the presence of sodium diclofenac or PPE (500 mg/kg) compared to the control group. This data indicates that PPE can increase postoperative analgesia in mice.

## Conclusions

From our study, it can be concluded that PPE possesses significant antinociceptive and anti-inflammatory effects in both chemical and thermal-induced pain models. The antinociceptive activity of PPE is probably due to the presence of high amounts of phenolic compounds and flavonoids, including catechin hydrate, para-coumaric acid, vanillic acid, and syringic acid. PPE has been found to be effective in alleviating pain after sciatic nerve surgery in mice. Further explorations are required to determine the long-term use of PPE in postoperative pain management. Nevertheless, we propose that PPE could be potential for the discovery and development of newer analgesic and anti-inflammatory drug which may also act as postoperative analgesic.

## Data Availability

The datasets used and/or analyzed during the current study are available from the first author on reasonable request.

## Abbreviations

PPE: Aqueous panch phoron extract
ARRIVE: Animal Research Reporting In Vivo Experiments
WHO: World Health Organization
IASP: International Association for the Study of Pain
NSAIDs: Nonsteroidal Anti-inflammatory Drugs
HPLC: High Performance Liquid Chromatography
IACUC: NSU Institutional Animal Care and Use Committee
MgCl_2_: Magnesium chloride
HCl: Hydrochloric acid
PGE2: Prostaglandin E2
PGF2α: Prostaglandin F2α
TNF-α: Tumor necrosis factor-α
IL-1β: Interleukin-1β
IL-8: Interleukin-8
SPSS: Statistical Package for the Social Sciences
ANOVA: Analysis of Variance
